# Immunological Features of Pediatric Interstitial Pneumonia Due to *Mycoplasma pneumoniae*

**DOI:** 10.3389/fped.2021.651487

**Published:** 2021-04-20

**Authors:** Xuefeng Xu, Yuanjian Sheng, Li Yang, Haichun Zhou, Lanfang Tang, Lizhong Du

**Affiliations:** ^1^Department of Rheumatology Immunology & Allergy, National Clinical Research Center for Child Health, The Children's Hospital, Zhejiang University School of Medicine, Hangzhou, China; ^2^Department of Pulmonary Medicine, National Clinical Research Center for Child Health, The Children's Hospital, Zhejiang University School of Medicine, Hangzhou, China; ^3^Department of Radiology, National Clinical Research Center for Child Health, The Children's Hospital, Zhejiang University School of Medicine, Hangzhou, China; ^4^Department of Neonatology, National Clinical Research Center for Child Health, The Children's Hospital, Zhejiang University School of Medicine, Hangzhou, China

**Keywords:** children, interstitial pneumonia, lymphocyte subtypes, *Mycoplasma pneumoniae*, cytokine

## Abstract

**Background:** Inflammatory response, oxidative stress, and immunologic mechanism are involved in the pathogenesis of *Mycoplasma pneumoniae* pneumonia (MPP). However, the role of immune system of pediatric interstitial pneumonia due to *M. pneumoniae* infections remains poorly understood. The aim of this study was to analyze the immunologic features of pediatric interstitial pneumonia due to *Mycoplasma pneumoniae (M. pneumoniae)*.

**Methods:** A retrospective study was conducted on a primary cohort of children with MPP. Propensity score analysis was performed to match interstitial pneumonia and pulmonary consolidation children.

**Results:** The clinical characteristics strongly associated with the development of interstitial pneumonia were boys, age >5 years, wheezing history, hydrothorax free, lymphocytes (>3.0 × 10^9^/L), CD19^+^ (>0.9 × 10^9^/L), CD3^+^ (>2.5 × 10^9^/L), CD4^+^ (>1.5 × 10^9^/L), CD8^+^ (>0.9 × 10^9^/L), interleukin-6 (IL-6, <30 pg/ml), IL-10 (<6 pg/ml), and interferon-γ (IFN-γ, <15 pg/ml). After propensity score analysis, children with interstitial pneumonia showed significantly higher CD19^+^, CD3^+^, and CD4^+^ T cell counts, and lower serum IL-6, IL-10, and IFN-γ levels. The final regression model showed that only CD4^+^ T cells (>1.5 × 10^9^/L, OR = 2.473), IFN-γ (<15 pg/ml, OR = 2.250), and hydrothorax free (OR = 14.454) were correlated with the development of interstitial pneumonia among children with MPP.

**Conclusions:** The *M. pneumoniae*-induced interstitial pneumonia showed increased CD4^+^ T cells and lower serum IFN-γ level. Specific immunologic profiles could be involved in the development of pediatric interstitial pneumonia due to *M. pneumoniae* infections.

## Introduction

Community-acquired pneumonia (CAP) is a significant cause of morbidity and mortality worldwide and a major public health threat to children in China (1). *Mycoplasma pneumoniae* (*M. pneumoniae*) is one of the main pathogens causing CAP in young adults and children (2). *Mycoplasma pneumoniae* pneumonia (MPP) accounts for up to 40% or more of CAP in children, increasing the rate of morbidity, mortality, as well as the cost of health care in China (3–6). Although MPP is usually a benign and self-limited disease, a number of cases were reported to develop into refractory MPP (3, 7–10). Children with refractory MPP frequently presented with delayed radiographic resolution and long-standing pulmonary sequelae such as bronchiectasis and atelectasis, even causing plastic bronchitis or requiring intensive care (3, 7, 11, 12). In addition to causing diseases in the respiratory system, *M. pneumoniae* has been implicated in several extrapulmonary complications including arthritis, liver injury, encephalitis, hemolytic anemia, and glomerulonephritis (10, 13). Furthermore, the development of extrapulmonary complications could be strongly associated with immune response induced by *M. pneumoniae* (14).

Advances in imaging diagnostic techniques have provided an opportunity to better study pulmonary diseases. MPP patients may present with interstitial pneumonia (IP), bronchopneumonia, and pulmonary consolidation (PC) or airspace consolidation based on the high-resolution computed tomography (HRCT) images (15). In addition to bronchopneumonia, children with MPP can also manifest IP and PC, especially in children older than 5 years (16, 17). Furthermore, children of the same age could present with either IP or PC. However, there is limited information in the literature about the development of IP in *M. pneumoniae* infections.

There is evidence that inflammatory response, oxidative stress reaction, cytokine imbalance, and immunologic reaction are involved in the pathogenesis of *M. pneumoniae* infections (13, 18–20). A specific cytokine pattern of moderately elevated interleukin-6 (IL-6), IL-10, and interferon-γ (IFN-γ) indicated a higher prediction of MPP among CAP patients (20). Furthermore, increased IFN-γ levels were strongly associated with the development of complications including pleural effusion and atelectasis in children with MPP. Given the role of cytokines in children with MPP, we hypothesized that immune dysfunction may contribute to the development of IP. The aim of the present study was to evaluate immunologic features of pediatric IP due to *M. pneumoniae* infections.

## Methods

### Patients and Data Collections

A retrospective study was conducted on a primary cohort of patients with refractory pneumonia underwent high-resolution computed tomography (HRCT) between January 2015 and December 2017 in the Children's Hospital, Zhejiang University School of Medicine. This retrospective study was approved by the Ethic Review Board of Children's Hospital, Zhejiang University School of Medicine (2019-IRB-104). Two pediatric radiologists examined the chest HRCT taken during hospitalization, and a consensus of interpretation was reached. The observers evaluated the patterns and distribution of lung parenchymal mediastinal, and pleural abnormalities. The patterns of pneumonia were also divided into three main categories on the basis of HRCT according to the previous studies: (15, 17, 21, 22). (1) Areas of increased pulmonary opacity with obscuration of underlying bronchovascular structures are defined as PC with or without air bronchograms; (2) a consolidation following bronchovascular branching or increased nodular densities along the bronchial trees were considered as a typical bronchopneumonic change (bronchopneumonia); (3) focal or diffused bronchovascular thickening without airspace opacity was considered interstitial pneumonia (IP), including bronchial wall thickness, ground glass opacity, tree-in-bud, reticular & linear opacity, nodular opacity, pleural thickness, interlobular septal thickness, and bronchiectasis. When at least two different patterns of pneumonia were observed in different lobes of the lungs, the pneumonia was classified as mixed pneumonia. In the present study, we collected the data on children with pneumonia who underwent HRCT, and those who did not perform HRCT were not included.

Diagnosis of MPP was based on diagnosis of CAP and etiology using our previous studies (20, 23). Briefly, CAP was diagnosed by clinical symptoms, signs, laboratory test, and chest radiographs. Current infection with *M. pneumoniae* was based on ≥4-fold changes in antibody titers between paired acute and convalescent sera, or positive IgM antibody (ELISA, Shanghai B&C Biological Technology, Co. Ltd., China), and positive *M. pneumoniae* DNA in nasopharyngeal aspirates or bronchoalveolar lavages based on the Taq-Man PCR technology (PCR fluorescence probing, Da An Gene Co., Ltd. of Sun Yat-sen University, China). Children with MPP who had co-infections, such as bacterial infections, Epstein-Barr virus, or respiratory syncytial virus infections were excluded. Those patients with tuberculosis or HIV positive were also excluded from the study, and those with chronic lung diseases, congenital heart disease, cerebral palsy, or tumor were also excluded. Demographic and clinical data were collected, including age, sex, duration of fever, imaging examination, and ultrasonography. All laboratory tests for collection were performed on admission.

### Cytokines and Lymphocyte Subtypes

For patients with pneumonia, cytokine detection, and lymphocyte subtyping were routinely performed. Venous blood samples were collected within 24 h of admission. The serum was isolated, and measurements of cytokines were performed by the FACScalibur^TM^ flow cytometry (Becton Dickinson, San Jose, CA, USA) immediately. Levels of interleukin-2 (IL-2), IL-4, IL-6, IL-10, tumor necrosis factor-α (TNF-α), and interferon-γ (IFN-γ) were quantitatively determined with the cytometric bead array (CBA) kit (CBA Human Th1/Th2 Cytokine Kit; BD Biosciences, San Jose, CA, USA) as described previously (20, 24). The BD CBA Software (BD Biosciences, San Jose, CA) was used to display the results in tabular and graphical format, and the standard curve was established for each individual set of reagents. The minimal and maximum limits of detections for these cytokines were 1.0 and 5,000 pg/mL, respectively. Lymphocyte subtype percentages were performed using BD Multitest 6-color TBNK Reagent via the Cantoll (Becton Dickinson, San Jose, CA, USA), and analyzed using DIVA software (Becton Dickinson, San Jose, CA). Lymphocyte subtype counts were calculated by multiplying the absolute value of lymphocytes and subtype percentages.

### Statistical Analysis

Continuous variables were expressed as mean and standard deviation (SD), and compared using an unpaired, 2-tailed *t* test or Mann-Whitney test. Categorical variables were compared using a Chi-Square test or Fisher exact test. The logistic regression analysis was used to assess potential risk factors of the development of IP due to *M. pneumoniae* infections. The area under the receiver operating characteristic (ROC) curve is developed to evaluate the predictive power of logistic regression model. The optimal cutoff values were determined by maximizing the Youden index (sensitivity + specificity−1). To exclude the impacts of age and gender, a propensity score analysis (PSA) was used to account for the baseline differences in the probability between children with IP and PC. IP and PC children with the same propensity score will have similar distributions of observed baseline covariates. R statistical software packages (R version 3.4.3) were used to perform all the statistical analysis and graphics, and *P* < 0.05 was considered statistically significant.

## Results

### Clinical Characteristics of MPP Children With IP

Nine hundred and seventy-seven patients were identified as HRCT-confirmed MPP. Patients with co-infection, other diseases, bronchopneumonia, or mixed pneumonia (*n* = 385), and those with missing values (24 IP and 79 PC) were excluded. Finally, 103 children with IP and 386 children with PC were included in the present study. The clinical characteristics of the two groups were listed in Table 1. The boy to girl ratio in children with IP was 62.1%, significantly higher than that (46.9%, *P* = 0.006) in children with PC. Children with IP had increased frequent wheezing (29.1 vs. 11.7%), higher lymphocyte counts (3.95 × 10^9^/L vs. 2.59 × 10^9^/L), and younger age (4.89 vs. 5.97 years) compared with children with PC. The average durations from symptom onset to hospital admission were 9.82 and 8.89 days in IP and PC children, respectively (Table 1). However, no significant statistical difference was observed between the two groups.

**Table 1:** Clinical characteristics and laboratory findings between IP and PC.

**Variable**	**Interstitial pneumonia**	**Pulmonary consolidations**	***P***
	**(*n* = 103)**	**(*n* = 386)**	
Gender (M/F)	64/39	181/205	0.006^**^
Age (year)	4.89 ± 3.23	5.97 ± 2.70	0.002^**^
Age (>5 year, *n*)	43 (41.7%)	231 (59.8%)	0.001^**^
Cesarean section (*n*)	54 (52.4%)	200 (51.8%)	0.912
Wheezing history (*n*)	30 (29.1%)	45 (11.7%)	<0.001^**^
Fever (day)	6.23 ± 4.56	6.32 ± 3.13	0.833
Duration from symptom onset to admission (day)	9.82 ± 4.87	8.89 ± 4.18	0.079
WBC counts (x10^9^/L)	9.00 ± 3.85	7.76 ± 3.63	0.002^**^
Neutrophil (%)	56.12 ± 16.89	64.73 ± 14.53	<0.001^**^
Eosinophils	0.98 ± 0.16	1.19 ± 0.11	0.334
Lymphocytes (x10^9^/L)	3.95 ± 2.57	2.59 ± 1.41	<0.001^**^
Hgb (g/L)	125.17 ± 11.21	120.87 ± 10.58	0.001^**^
PLT (x10^9^/L)	326.89 ± 114.83	279.10 ± 95.80	<0.001^**^
CRP (0–8 mg/L)	16.65 ± 2.25	42.51 ± 2.18	<0.001^**^
PCT (0–0.46 ng/mL)	0.42 ± 0.31	0.55 ± 0.09	0.585
IgG (6.36–14.04 g/L)	9.47 ± 2.47	9.59 ± 3.19	0.723
IgA (0.63–1.79 g/L)	1.08 ± 0.69	1.19 ± 0.64	0.117
IgE (0–100 IU/mL)	210.06 ± 23.74	227.25 ± 14.67	0.579
Pleural effusion (*n*)	4 (3.9%)	167 (43.3%)	<0.001^**^

*CRP, C reactive protein; Hgb, hemoglobin; IFN, interferon; Ig, Immunoglobulin; IL, interleukin; PCT, procalcitonin; PLT, blood platelet; WBC, white blood cells*.

** *P < 0.01*.

### Cellular Immunologic Features and Serum Immunoglobulin Levels

Although there were no significant differences in humoral immunity including serum IgG (9.47 vs. 9.59, *P* = 0.923), IgA (1.08 vs. 1.19, *P* = 0.117), and IgE (210.06 vs. 227.25, *P* = 0.579) levels between IP and PC groups, serum IgE levels in MPP were more than twice the upper limit of normal reference ranges (Table 1). Moreover, about half of IP children (52%) had a high IgE level with >100 IU/mL, similar to PC children (48%, *P* = 0.41). Patients with IP demonstrated higher lymphocytes (3.95 × 10^9^/L vs. 2.59 × 10^9^/L, *P* < 0.001), including higher CD19^+^ T cells (0.92 × 10^9^/L vs. 0.51 × 10^9^/L, *P* < 0.001), CD3^+^ T cells (2.58 × 10^9^/L vs. 1.69 × 10^9^/L, *P* < 0.001), CD4^+^ T cells (1.43 × 10^9^/L vs. 0.90 × 10^9^/L, *P* < 0.001), and CD8^+^ T cells (0.91 × 10^9^/L vs. 0.56 × 10^9^/L, *P* < 0.001) compared with those with PC (Table 1). Similar to our previous study (20), the present study also showed increased serum IL-6, IL-10, and IFN-γ levels in patients with IP or PC. However, children with IP had lower serum levels of IL-6 (28.29 vs. 63.97 pg/ml, *P* < 0.001), IL-10 (5.88 vs. 8.59 pg/ml, *P* < 0.001), IFN-γ (14.55 vs. 43.29 pg/ml, *P* < 0.001) relative to children with PC.

### Risk Factors of the Development of IP

**Figure 1:**
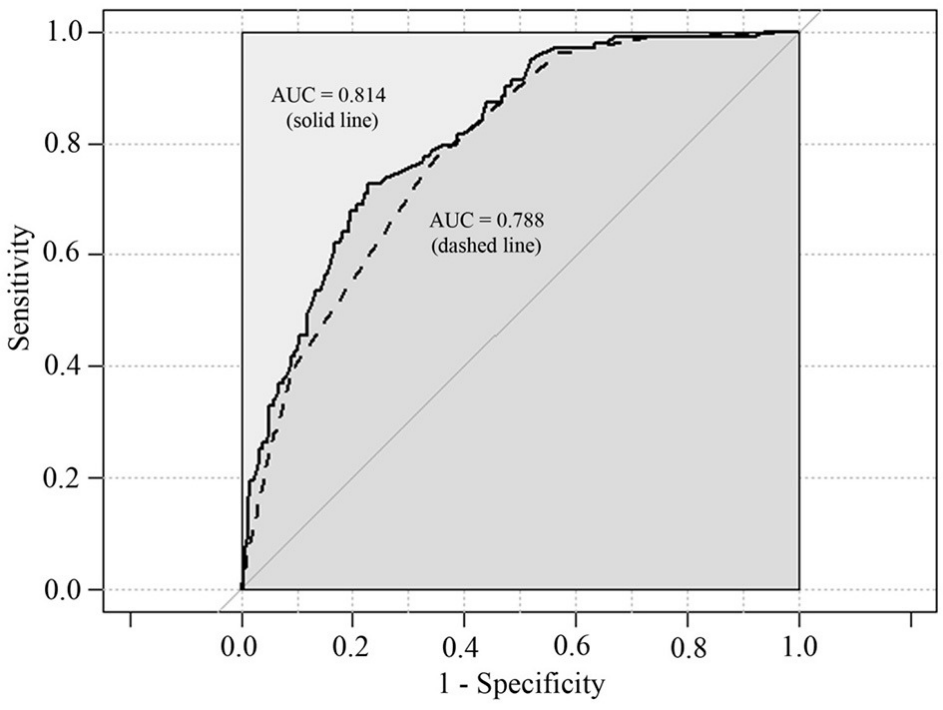
Predictive ability of multivariate logistic regression models for IP. Area under the curves (AUC) for solid line is 0.814 (95% CI 0.771–0.857, *P* < 0.001). AUC for dashed line is 0.788 (95% CI 0.743–0.832, *P* < 0.001).

The univariate logistic regression models for prediction of IP showed there were significant differences in most of potential risk factors (Table 2). The clinical characteristics most strongly associated with the development of IP were boys, age >5 years, wheezing history, hydrothorax free, lymphocyte counts (>3.0 × 10^9^/L), CD19^+^ (>0.9 × 10^9^/L), CD3^+^ (>2.5 × 10^9^/L), CD4^+^ (>1.5 × 10^9^/L), CD8^+^ (>0.9 × 10^9^/L), IL-6 (<30 pg/ml), IL-10 (<6 pg/ml), and IFN-γ (<15 pg/ml) compared with children with PC. Multivariate logistic regression model indicated that only wheezing history, boys, hydrothorax free, and IFN-γ <15 pg/ml were independent predictors. The area under the curve (AUC) for this model was 0.814 (Figure 1, solid line, 95% CI 0.771–0.857, *P* < 0.001).

**Table 2:** Risk factors associated with the development of IP on logistic regression analysis.

**Variables**	**Univariate logistic regression**				**Multivariate logistic regression**			
	**B efficient**	**OR**	**95% CI**	***P***	**B efficient**	**OR**	**95% CI**	***P***
Gender (boys vs. girls)	0.620	1.859	1.190–2.902	0.006^**^	0.591	1.805	1.090–2.988	0.022^*^
Age > 5 vs. ≤ 5 years	−0.732	0.481	0.309–0.748	0.001^**^	−0.025	0.975	0.542–1.754	0.933
Wheezed history (yes)	1.136	3.114	1.839–5.273	<0.001^**^	0.789	2.200	1.205–4.017	0.010^*^
Hydrothorax (no)	2.938	18.873	6.808–52.322	<0.001^**^	2.599	13.444	4.720–38.295	<0.001^**^
Lymphocytes (> 3.0 vs. ≤ 3.0 × 10^9^/L)	1.082	2.949	1.886–4.611	<0.001^**^	0.521	1.684	0.737–3.848	0.216
CD3^−^CD19^+^ T cells (> 0.9 vs. ≤ 0.9 × 10^9^/L)	1.378	3.967	2.346–6.709	<0.001^**^	0.349	1.418	0.650–3.091	0.380
CD3^+^ T cells (> 2.5 vs. ≤ 2.5 × 10^9^/L)	1.089	2.971	1.810–4.875	<0.001^**^	−0.463	0.629	0.212–1.870	0.405
CD3^+^CD4^+^ T cells (> 1.5 vs. ≤ 1.5 × 10^9^/L)	1.418	4.128	2.417–7.048	<0.001^**^	0.761	2.141	0.801–5.722	0.129
CD3^+^CD8^+^ T cells (> 0.9 vs. ≤ 0.9 × 10^9^/L)	0.869	2.348	1.475–3.852	<0.001^**^	−0.241	0.786	0.345–1.792	0.567
IL-6 (≤ 30 vs. > 30 pg/ml, reference range 1.7–16.6 pg/ml)	0.835	2.305	1.407–3.776	<0.001^**^	0.208	1.231	0.588–2.578	0.581
IL-10 (≤ 6 vs. > 6 pg/ml, reference range 2.6–4.9 pg/ml)	0.607	1.834	1.183–2.843	0.007^**^	−0.126	0.881	0.449–1.730	0.714
IFN-γ (≤ 15 vs. > 15 pg/ml, reference range 1.6–17.3 pg/ml)	1.222	3.394	2.094–5.499	<0.001^**^	0.834	2.303	1.313–4.041	0.004^**^

*OR, odds ratio; CI, confidence interval*.

* *P < 0.05*,

** *P < 0.01*.

### Comparisons Between IP and PC Children After PSA

**Figure 2:**
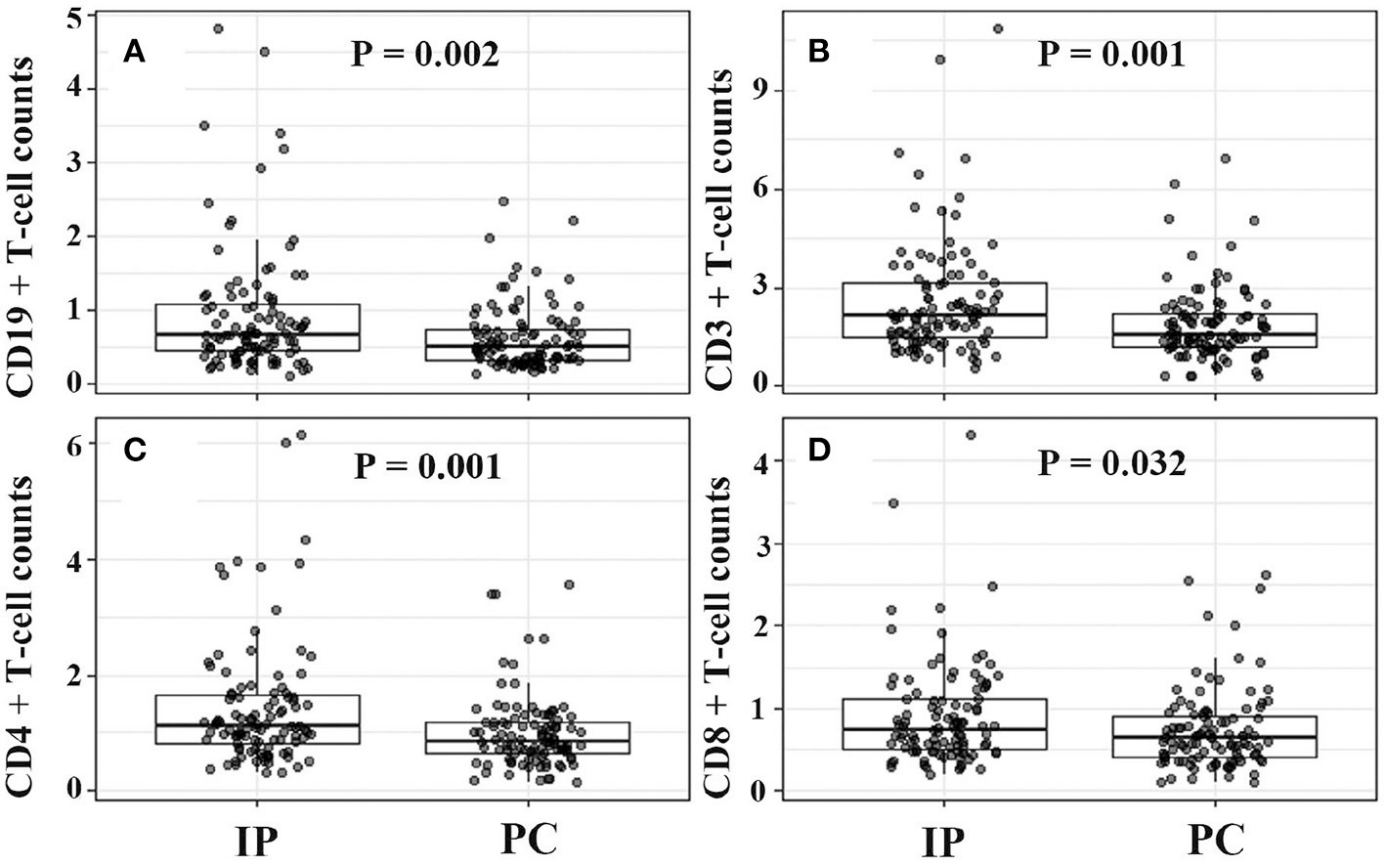
Increased CD19^+^, CD3^+^, CD4^+^, CD8^+^ T cell counts in IP children due to *Mycoplasma pneumoniae* infections. **(A–D)** Were for comparison between IP and PC children after PSA. IP, interstitial pneumonia; PC, pulmonary consolidation; PSA, propensity score analysis.

**Figure 3:**
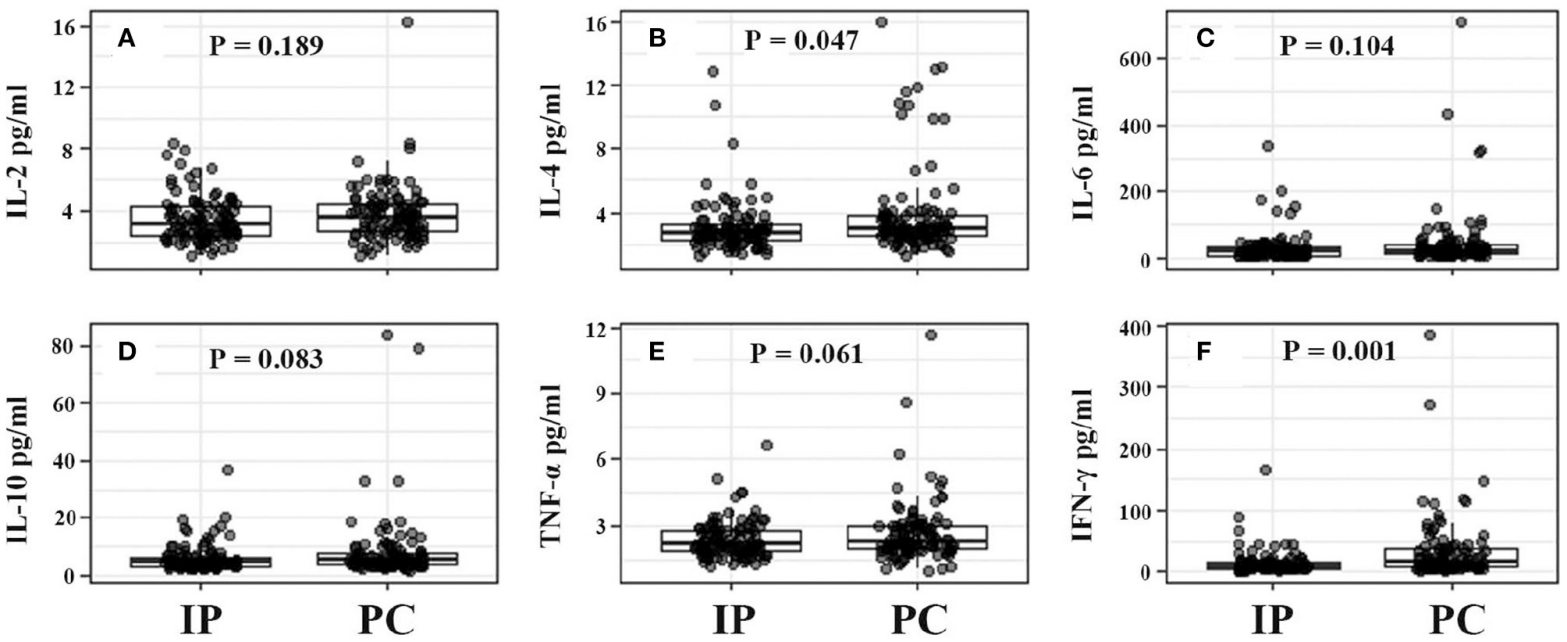
Serum cytokine levels from IP and PC children. **(A–F)** Were for comparison between IP and PC children after PSA. IFN, interferon; IP, interstitial pneumonia; PC, pulmonary consolidation; PSA, propensity score analysis; TNF, tumor necrosis factor.

In view of the significant differences in patient's age and gender ratio, we performed propensity score analysis (PSA) to match IP children with PC children (1:1 matching). Compared with age-matched children with PC, children with IP had more frequent history of wheezing (29.1 vs. 16.5%), and higher lymphocyte counts (3.95 × 10^9^/L vs. 2.99 × 10^9^/L). Furthermore, patients with IP showed significantly higher CD19^+^ T cell counts (0.92 × 10^9^/L vs. 0.61 × 10^9^/L), CD3^+^ T cell (2.58 × 10^9^/L vs. 1.87 × 10^9^/L), and CD4^+^ T cell counts (1.43 × 10^9^/L vs. 1.00 × 10^9^/L), and lower serum IL-6 (28.29 vs. 44.72 pg/ml), IL-10 (5.88 vs. 8.05 pg/ml), and IFN-γ (14.55 vs. 32.97 pg/ml) levels relative to PC children (Table 3 and Figures 2, 3).

**Table 3:** Differences in clinical characteristics between IP and PC after propensity score analysis (PSA).

**Variable**	**Interstitial**	**Pulmonary**	***P***
	**pneumonia**	**consolidations**	
	**(*n* = 103)**	**(*n* = 103)**	
Gender (M/F)	64/39	64/39	
Age (year)	4.89 ± 3.23	4.86 ± 2.58	0.951
Age (>5 years, *n*)	43	43	
Cesarean section (*n*)	54 (52.4%)	59 (57.3%)	0.484
Wheezed history (*n*)	30 (29.1%)	17 (16.5%)	0.031^*^
Fever (day)	6.23 ± 4.56	5.96 ± 3.49	0.644
WBC counts (x10^9^/L)	9.00 ± 3.85	8.39 ± 3.70	0.245
Neutrophil (%)	56.12 ± 16.89	63.08 ± 14.76	0.002^**^
Eosinophils	0.98 ± 0.16	1.45 ± 0.28	0.142
Lymphocytes (x10^9^/L)	3.95 ± 2.57	2.99 ± 1.70	0.002^**^
Hgb (g/L)	125.17 ± 11.21	122.87 ± 9.61	0.115
PLT (x10^9^/L)	326.89 ± 114.83	291.42 ± 99.64	0.019^*^
CRP (0–8 mg/L)	16.65 ± 2.25	38.99 ± 4.18	<0.001^**^
PCT (0–0.46 ng/mL)	0.42 ± 0.31	0.60 ± 0.23	0.631
IgG (6.36–14.04 g/L)	9.47 ± 2.47	9.32 ± 2.83	0.684
IgA (0.63–1.79 g/L)	1.08 ± 0.69	1.01 ± 0.58	0.399
IgE (0–100 IU/mL)	210.06 ± 23.74	238.43 ± 29.78	0.457
Pleural effusion (n)	4 (3.9%)	39 (37.9%)	<0.001^**^

*CRP, C reactive protein; Hgb, hemoglobin; IFN, interferon; Ig, Immunoglobulin; IL, interleukin; PCT, procalcitonin; PLT, blood platelet; WBC, white blood cells*.

* *P < 0.05*,

** *P < 0.01*.

**Figure 4:**
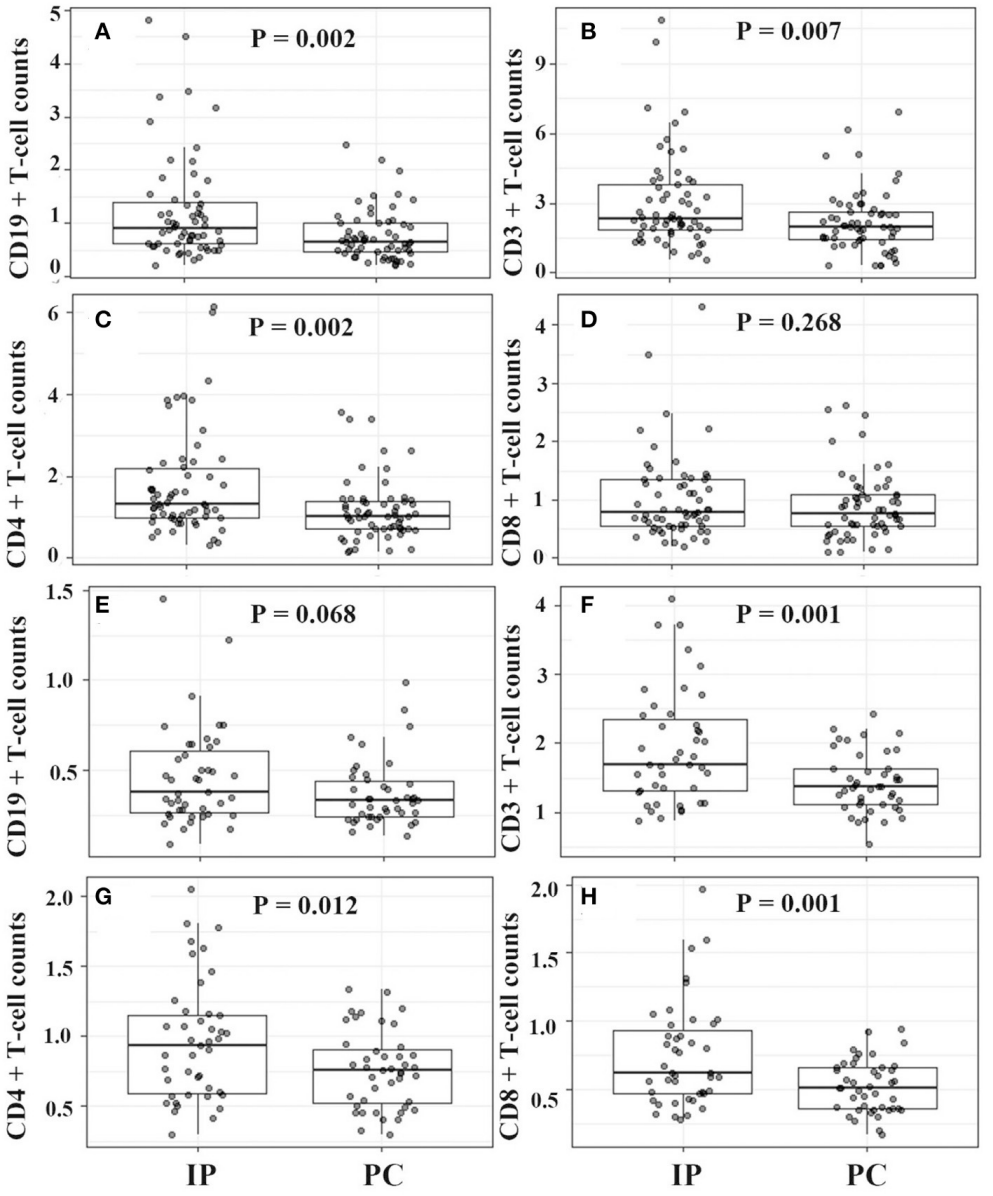
Lymphocyte classification counts between IP and PC children ≤5 years **(A–D)** and >5 years **(E–H)** after PSA. IP, interstitial pneumonia; PC, pulmonary consolidation; PSA, propensity score analysis.

**Figure 5:**
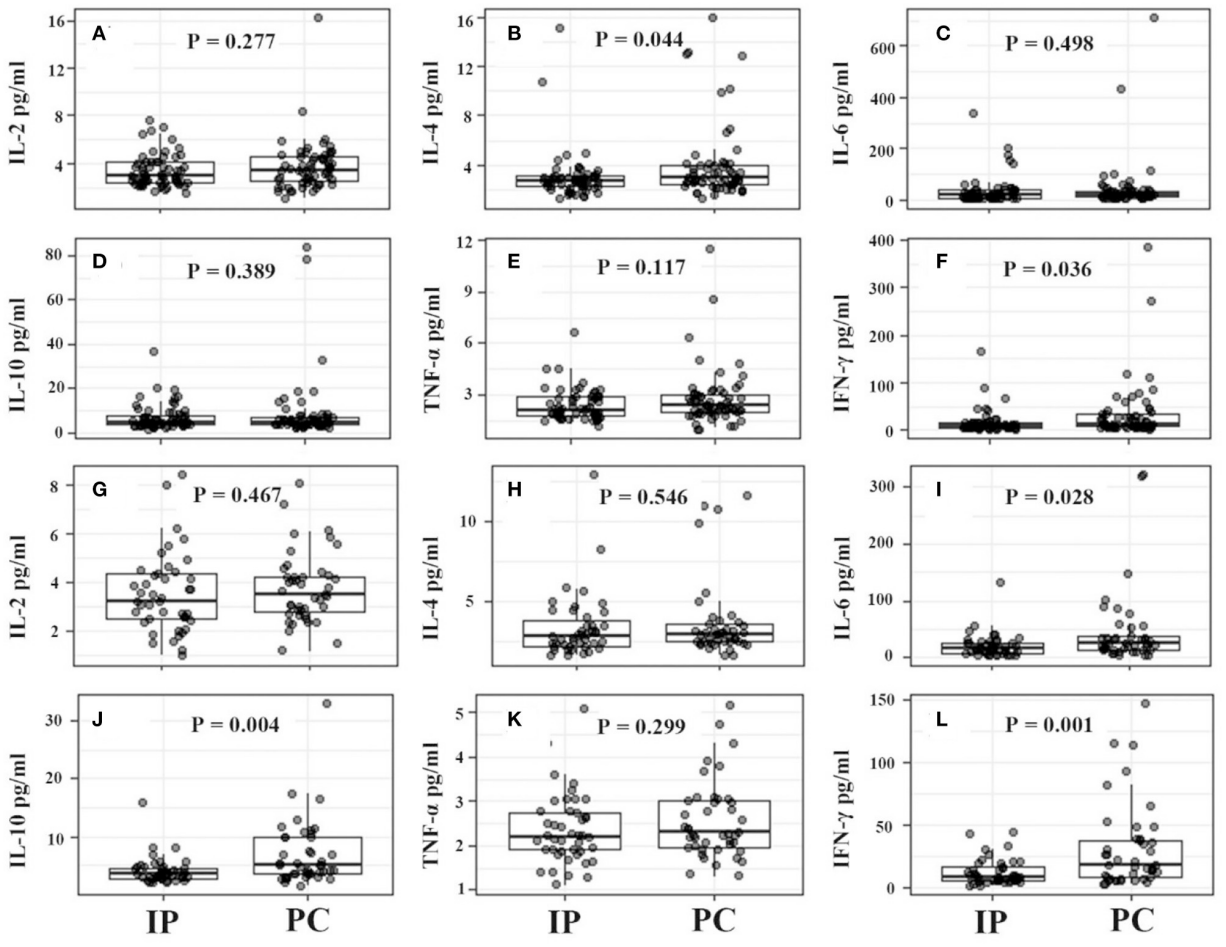
Serum cytokine levels between IP and PC children ≤5 years **(A–F)** and >5 years **(G–L)** after PSA. IFN, interferon; IP, interstitial pneumonia; PC, pulmonary consolidation; PSA, propensity score analysis; TNF, tumor necrosis factor.

For IP children with ≤5 years, they showed significantly higher counts of lymphocytes, CD19^+^ T cells, CD3^+^ T cells, and CD4^+^ T cells, and much lower serum IFN-γ levels (Figures 4, 5). However, for IP children with >5 years, they demonstrated higher counts of lymphocytes, CD3^+^ T cells, CD4^+^ T cells, and CD8^+^ T cells, and lower serum IL-6, IL-10, and IFN-γ compared with age-matched PC children (Figures 4, 5). In addition, IP children were much less likely to have hydrothorax regardless of age.

### Role of Immunologic Response in IP

The abovementioned PSA result revealed that increased blood lymphocyte counts, CD3^+^ T cells, CD4^+^ T cell counts, lower serum IFN-γ level, and hydrothorax free were strongly associated with the development of IP. In the final multivariate logistic regression model, only CD4^+^ T cells (>1.5 × 10^9^/L, OR = 2.473, 95% CI: 1.011–6.046, *P* = 0.047), IFN-γ (<15 pg/ml, OR = 2.250, 95% CI: 1.326–3.817, *P* = 0.003), and hydrothorax free (OR = 14.454, 95% CI: 5.158–40.503, *P* < 0.001) were independent predictors. ROC curve graph revealed that this model could be used to predict the development of IP. The AUC was 0.788 (Figure 1, dashed line, 95% CI 0.743–0.832, *P* < 0.001).

## Discussion

*Mycoplasma pneumoniae* is one of the smallest self-replicating organisms capable of cell-free existence, causing respiratory diseases, and extrapulmonary complications. For children with the same age, MPP may present with either IP or PC. Our study indicated that there exists significant difference in lymphocyte counts and cytokine level between IP and PC children due to *M. pneumoniae*. The increased CD4^+^ T cells and decreased serum IFN-γ level contributed to the development of IP in children with *M. pneumoniae* infections.

In the present study, a univariate logistic regression showed that age, gender, wheezed history, hydrothorax, lymphocyte differential counts, and cytokine levels were significantly associated with the development of pediatric IP by *M. pneumoniae* infections. The multivariate regression model demonstrated that wheezing history, boy, hydrothorax free, and IFN-γ <15 ng/ml were significantly correlated with IP. In view of the impacts of age and gender, IP children were matched with PC children using propensity score analysis. Relative to PC children, IP children showed significantly higher CD19^+^ T cell counts, CD3^+^ T cell, and CD4^+^ T cell counts, and lower serum IL-6, IL-10, and IFN-γ levels. Further age stratification indicated that only CD3^+^ and CD4^+^ T cell counts had significant statistical significance between IP and PC children. In the final multivariate logistic regression model, only increased CD4^+^ T cells (>1.5 × 10^9^/L), decreased IFN-γ (<15 pg/ml), and hydrothorax free were independent predictors for the development of IP.

Increasing evidence shows that different interstitial lung disease had various ratios of lymphocyte subtypes regardless of from serum or bronchoalveolar lavage (BAL) samples (25, 26). A prevalence of CD4^+^ T cells in BAL is suggestive of sarcoidosis, whilst prevalence of CD8^+^ cells is suggestive of hypersensitivity pneumonitis (25). The different inflammatory cell profiles are a continuum, which depend on the extension and intensity of the inflammatory cell infiltration in the alveolar level (27). CD4^+^ T cells are critical for the development of interstitial pneumonitis followed by rheumatoid arthritis (28). An increased specific CD4^+^ T cell subtype (CD4+CXCR4+ T cell) was significantly associated with the severity and mortality of idiopathic inflammatory myopathy-associated interstitial lung disease (29). *Mycoplasma pneumoniae* immunized or P1 protein immunized mice had significantly increased CD4^+^ T cells and CD4^+^/CD8^+^ ratio in spleen cells (30). The subtype of CD4+ T cells (Th17 cells) was also significantly higher in *M. pneumoniae-*infected patients with extra-pulmonary manifestations (31). Furthermore, patients who experienced a short course of MPP showed a significant increase in the percentage of Th17 cells compared with those with a long course of MPP (31). Additionally, community-acquired respiratory distress syndrome toxin from *M. pneumoniae* was capable of inducing allergic-type inflammation in naïve animals, which is dependent on CD4^+^ T cells (32). These studies demonstrated that CD4^+^ T cells could play a major role in the development of IP among patients with MPP.

Our study also showed that *M. pneumoniae*-induced IP had significantly increased serum CD4^+^ T cells compared with those PC patients, which appeared to be like adenovirus pneumonia manifestations. For patients with adenovirus pneumonia, those without pleural effusion had significantly higher CD4^+^ T cell counts relative to those with pleural effusion (33). Additionally in chicken trachea with *Mycoplasma gallisepticum* infections, CD8^+^ T cells were clustered in follicular-like arrangements, while the distribution of CD4^+^ T cells was dispersed (34). Moreover, different from the fact that either *Streptococcus pneumoniae* or *Klebsiella pneumoniae* sonicated antigens markedly decreased viable lymphocyte counts, *M. pneumoniae* antigens promoted the proliferation of lymphocytes (35), further indicating that the predominance of mycoplasma-induced CD4^+^ T cell could possess species-specific nature. Altogether, it is likely that increased specific CD4^+^ T cells in *M. pneumoniae* infections were strongly associated with the development of IP.

Notably, there were obvious differences in serum cytokine levels between IP and PC children due to *M. pneumoniae* infections. Our previous study had found that moderately elevated IL-6, IL-10, and IFN-γ could predict *M. pneumoniae* infections among CAP patients (20). Moreover, increased serum IFN-γ levels were strongly associated with CAP severity (23). The present study demonstrated that the IP children had a lower level of serum IFN-γ than PC patients, suggesting a moderate inflammatory response among IP patients. On the other hand, the presence of hydrothorax often indicates a severe disease or a strong inflammatory response. Although hydrothorax could be associated with an increased IFN-γ level, IP children were much less likely to have hydrothorax (3.9%) compared with PC children (43.3%). This result also suggested that hydrothorax in IP patients had little effect on cytokine production. In general, IP children showed a lower inflammatory response than PC patients. Due to the limited availability of the commercial cytometric bead array kit, our hospital only detected IL-2, 4, 6, 10, TNF-α, and IFN-γ, combined detection of cytokines (e.g., IL-17) could have a greater power to predict the presence of IP.

There is evidence that serum IgE levels significantly increased in the atopic patients with MPP than in the nonatopic patients. Furthermore, MPP children with atopy were more likely to present with tachypnea, oxygen supplement, and steroid use (36). Additionally, the MPP patients with extra-pulmonary diseases demonstrated a significantly increased serum IgE (37). Our study also demonstrated an increased IgE level in refractory MPP, indicating that the presence of atopy may be associated with severe MP pneumonia. Although serum IgE levels from PC patients were slightly higher than those from IP patients, there was no significant difference between the two groups. In contrast, the proportion of hyper-IgE cases in IP patients were slightly more than that in PC children. However, no significant difference was observed between IP and PC children. This inconsistency between hyper-IgE values and cases in IP and PC patients complicated the pathogenesis of MPP. Whether this weak difference could be related to the development of IP still needs to be further investigated. Another common extra-pulmonary complication was hydrothorax in the present study, especially in PC children. Compared with the PC children, IP children were much less likely to have hydrothorax, suggesting that hydrothorax in IP patients had little effect on cytokine changes or lymphocyte counts.

Our study had several limitations. First, all the laboratory tests were performed within 24 h of admission without dynamic monitoring. Second, the imaging features of bronchopneumonia might be characterized by interstitial pneumonia and pitchy shadowing. To better study interstitial pneumonia, we excluded children with bronchopneumonia, which could lead to a selective bias. Finally, the present study was only based on our single institutional clinical data, and other specific markers (such as T17 cell counts, IL-18 level) might contribute to better understanding the pathogenesis of IP caused by *M. pneumoniae*.

## Conclusion

The present study showed that increased CD4^+^ T cells and lower serum IFN-γ level were associated with the development of pediatric IP by *M. pneumoniae* infections. Specific immunologic profiles (increased CD4^+^ T cells and lower serum IFN-γ level) could be strongly correlated with the presence of IP among children with MPP. A prospective and multicenter study on immunological response would contribute to better understanding the pathogenesis of pediatric IP caused by *M. pneumoniae* infections.

## Data Availability Statement

The raw data supporting the conclusions of this article will be made available by the authors, without undue reservation.

## Ethics Statement

The studies involving human participants were reviewed and approved by the Ethic Review Board of Children's Hospital, Zhejiang University School of Medicine. Written informed consent to participate in this study was provided by the participants' legal guardian/next of kin.

## Author Contributions

XX, YS, and LD designed the study, interpreted the data and critically reviewed and revised the manuscript. LT undertook data collection and critically reviewed and revised the manuscript. LY and HZ undertook data collection, contributed to analyses and data interpretation, drafted the initial manuscript, and critically reviewed and revised the manuscript. All authors contributed to the article and approved the submitted version.

## Conflict of Interest

The authors declare that the research was conducted in the absence of any commercial or financial relationships that could be construed as a potential conflict of interest.
